# Prevention of Rectal SHIV Transmission in Macaques by Daily or Intermittent Prophylaxis with Emtricitabine and Tenofovir 

**DOI:** 10.1371/journal.pmed.0050028

**Published:** 2008-02-05

**Authors:** J. Gerardo García-Lerma, Ron A Otten, Shoukat H Qari, Eddie Jackson, Mian-er Cong, Silvina Masciotra, Wei Luo, Caryn Kim, Debra R Adams, Michael Monsour, Jonathan Lipscomb, Jeffrey A Johnson, David Delinsky, Raymond F Schinazi, Robert Janssen, Thomas M Folks, Walid Heneine

**Affiliations:** 1 Division of HIV/AIDS Prevention, National Center for HIV/AIDS, Viral Hepatitis, STD, and TB Prevention, Centers for Disease Control and Prevention, Atlanta, Georgia, United States of America; 2 Division of Scientific Resources, National Center for Preparedness, Detection, and Control of Infectious Diseases, Centers for Disease Control and Prevention, Atlanta, Georgia, United States of America; 3 Emory University School of Medicine/Veterans Affairs Medical Center, Decatur, Georgia, United States of America; St. Vincent's Hospital, Australia

## Abstract

**Background:**

In the absence of an effective vaccine, HIV continues to spread globally, emphasizing the need for novel strategies to limit its transmission. Pre-exposure prophylaxis (PrEP) with antiretroviral drugs could prove to be an effective intervention strategy if highly efficacious and cost-effective PrEP modalities are identified. We evaluated daily and intermittent PrEP regimens of increasing antiviral activity in a macaque model that closely resembles human transmission.

**Methods and Findings:**

We used a repeat-exposure macaque model with 14 weekly rectal virus challenges. Three drug treatments were given once daily, each to a different group of six rhesus macaques. Group 1 was treated subcutaneously with a human-equivalent dose of emtricitabine (FTC), group 2 received orally the human-equivalent dosing of both FTC and tenofovir-disoproxil fumarate (TDF), and group 3 received subcutaneously a similar dosing of FTC and a higher dose of tenofovir. A fourth group of six rhesus macaques (group 4) received intermittently a PrEP regimen similar to group 3 only 2 h before and 24 h after each weekly virus challenge. Results were compared to 18 control macaques that did not receive any drug treatment. The risk of infection in macaques treated in groups 1 and 2 was 3.8- and 7.8-fold lower than in untreated macaques (*p* = 0.02 and *p* = 0.008, respectively). All six macaques in group 3 were protected. Breakthrough infections had blunted acute viremias; drug resistance was seen in two of six animals. All six animals in group 4 that received intermittent PrEP were protected.

**Conclusions:**

This model suggests that single drugs for daily PrEP can be protective but a combination of antiretroviral drugs may be required to increase the level of protection. Short but potent intermittent PrEP can provide protection comparable to that of daily PrEP in this SHIV/macaque model. These findings support PrEP trials for HIV prevention in humans and identify promising PrEP modalities.

## Abstract 2

Using a repeat-exposure macaque model, Walid Heneine and colleagues find that pre-exposure prophylaxis with combination antiretroviral drugs provides protection against rectal challenge with a SHIV virus.

## Editors' Summary

**Background.:**

Each year, some 2.5 million people become newly infected with HIV, the virus that causes AIDS. A vaccine that protects people against HIV infection is not likely to become available for at least several years. Condoms can prevent infection, but they are not 100% effective, and people do not always use them. Until a vaccine becomes available, other methods of preventing HIV could save many thousands of lives.

One possibility for preventing HIV is pre-exposure prophylaxis (PrEP). In this approach, people who are likely to be exposed to the virus could take medicine to prevent them from becoming infected, in the same way that medication to protect against malaria is often taken by people traveling to high-risk areas.

**Why Was This Study Done?:**

PrEP has never been shown to be effective against sexual transmission of HIV in humans. Studies of PrEP in people at risk for HIV are currently in progress or being planned, but it is not clear which medications or dosing schedules should be used. The researchers in this study wanted to explore several possible ways of giving PrEP in monkeys in order to learn more about how to design strategies for testing PrEP in humans.

**What Did the Researchers Do and Find?:**

The researchers simulated human exposure to HIV by exposing rhesus macaques (a type of monkey) to SHIV, a monkey virus (SIV) that has been modified to contain the same outer protein as HIV. They exposed the macaques to repeated low doses of SHIV given rectally once per week, to simulate a common route of HIV transmission in humans. They used five groups of macaques that were all given the same viral exposure, but received different PrEP regimens: one group of six animals received only the anti-HIV drug emtricitabine (FTC), by injection under the skin every day; another six received FTC in combination with an oral form of the anti-HIV drug tenofovir every day, both by mouth; six received by injection FTC in combination with a higher tenofovir dose every day, and six also received by injection FTC in combination with the high-dose tenofovir, but the treatment was given only before and after the weekly viral exposure instead of every day. For comparison, the fifth group of nine macaques (plus another nine from a previous study) received no anti-HIV medications.

The researchers found that the macaques in any of the four treatment groups were less likely to become infected than those in the comparison group. In particular, all of the macaques receiving both FTC and high-dose tenofovir, whether daily or only around the time of exposure, were protected from infection. Macaques that did become infected in the other treatment groups had lower levels of virus in their blood than those that became infected in the comparison group, but some that became infected in the treatment groups went on to develop virus that was resistant to FTC.

**What Do These Findings Mean?:**

These results show that PrEP can be effective in this animal model, and that higher doses and combination treatments may be more effective than single drugs or lower doses. The results also suggest that PrEP might work if taken only around the time of exposure and therefore might not need to be taken every day in order to be effective. Further, by reducing the levels of HIV in people who do become infected, PrEP might reduce the chance that these people will transmit HIV to others before realizing that they themselves are infected. However, this study also demonstrates that partially effective PrEP can result in infection with drug-resistant virus, which might make treatment difficult. Also, the doses of tenofovir used to achieve maximum protection in these macaques may be higher than would be safe in humans. Carefully designed human studies will be needed to determine which, if any, PrEP strategies will be effective in practice.

**Additional Information.:**

Please access these Web sites via the online version of this summary at http://dx.doi.org/doi:10.1371/journal.pmed.0050028.

- The US Centers for Disease Control and Prevention (CDC) is conducting several trials of PrEP
- The AIDS Vaccine Advocacy Coalition and the UCLA Program in Global Health provide information at PreP Watch
- The UCSF Center for HIV Information's HIV InSite includes resources on HIV prevention and treatment

## Introduction

With an estimated 33.2 million people worldwide living with HIV at the end of 2007 and an estimated 2.5 million new infections in 2007 acquired mostly through sex, HIV/AIDS continues to be a major global health challenge [1]. Currently, no vaccine is available to prevent HIV, and it is unlikely that one will be developed soon. Pre-exposure prophylaxis (PrEP) with antiretroviral drugs is gaining considerable attention as a possible biomedical intervention strategy to prevent sexual transmission of HIV [2–5]. PrEP is a proven concept for other infectious diseases like malaria. Mathematical models estimate that over the next 10 y, an effective PrEP program could prevent 2.7 to 3.2 million new HIV-1 infections in sub-Saharan Africa [6]. This potentially significant public health benefit requires a very high PrEP efficacy and might be lost or substantially reduced with a PrEP efficacy of <50%. Therefore, identifying highly effective PrEP modalities is critical. In sexual transmission, HIV first replicates at a low level at the mucosal point of entry in the new host [7,8]. Effective PrEP can exploit this brief period of virus vulnerability by blocking HIV from establishing itself as a persistent infection.

Simian immunodeficiency virus (SIV) infection of macaques is a well-established model for HIV transmission that can provide data about the relative efficacy of different PrEP strategies, thereby informing the development of PrEP trials in humans. The use in PrEP of drugs that are approved for the treatment of HIV-1-infected persons is advantageous because of the known antiviral activity, safety, and drug resistance profiles of these drugs. Previous work in macaque models has largely focused on single-drug PrEP with tenofovir, a nucleotide reverse transcriptase (RT) inhibitor, and has shown the prophylactic efficacy of this drug against SIV transmission [9–11]. More recent work with tenofovir disoproxil fumarate (TDF), the currently approved oral prodrug, showed that daily PrEP with TDF at dosing comparable to the human dose was only partially protective against rectal or oral SIV/SHIV transmission [12,13]. In a recently reported Phase II clinical trial with TDF, two PrEP failures were reported compared to six infections in the placebo group [14]. The efficacy of TDF could not be conclusively evaluated because of the small number of HIV infections observed during the trial.

In this study, we evaluated PrEP efficacy of regimens containing the nucleoside RT inhibitor emtricitabine (FTC) alone or in combination with either tenofovir (in subcutaneous regimens) or TDF (in oral regimens). FTC is well tolerated and very potent, and has synergistic to additive effects with tenofovir. Both drugs have long intracellular half-lives and are co-formulated in a once-daily pill (Truvada) [15–18]. PrEP efficacy was measured in a repeat-low-dose exposure model in macaques that is highly relevant to PrEP in humans in three major ways. First, the SHIV virus challenge contains an R5 tropic HIV-1 envelope that resembles naturally transmitted viruses. Second, the mucosal transmission component uses a lower and more physiologic virus inoculum than what is conventionally used in the single high-dose challenge models [19,20]. Third, while single-dose models measure protection against one transmission event per animal, this model evaluates protection against multiple transmission events in each animal resulting from repeated virus exposures. In this model, protection by PrEP is measured by the degree that infection is prevented or by the increase in the number of exposures needed to infect macaques.

## Methods

### Animals and Interventions

The Animal Care and Use Committee (AICUC) of the Centers for Disease Control and Prevention (CDC) approved this study (protocol CDC-AICUC 1414OTTMONC-A5). All animal procedures were done at the animal facilities at CDC. Results reported and analyzed adhere to the predetermined study design and objectives.

**Figure 1:**
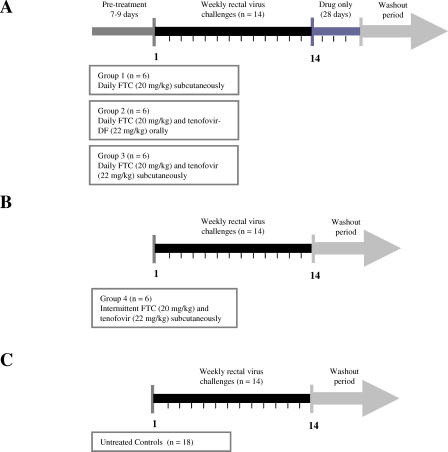
Study Design and Interventions (A) Daily PrEP. Macaques were drug-treated 7–9 d before the first virus inoculation. Treated animals that remained negative during the 14 challenges received drug for an additional 28 d. Treated animals that became infected continued treatment to monitor plasma viremia and drug resistance emergence. (B) Intermittent PrEP. Macaques received FTC and tenofovir only 2 h before and 24 h after each weekly rectal challenge. (C) Untreated control macaques. Infection of macaques was monitored weekly by PCR and serologic testing. The treatment groups were staggered; four of 33 macaques were used in two separate arms (see Methods, Text S1, and Table S1).

The study design is shown in Figure 1. Three drug treatments with different antiviral activity were each given once daily to a group of six rhesus macaques. Group 1 was treated subcutaneously with a human equivalent dose of FTC (20 mg/kg; see next paragraph). Group 2 received oral FTC and TDF (20 mg/kg and 22 mg/kg, respectively) at a dose equivalent to Truvada in humans. Group 3 received subcutaneous FTC (20 mg/kg) and a higher dose of tenofovir (22 mg/kg). Based on approximately double the molecular weight and a 25%–50% tenofovir bioavailability in macaques following oral TDF administration, this dose corresponds to about 66–88 mg/kg of TDF given orally, or 3- to 4-fold the human-equivalent dosing. Intermittent PrEP was given to a fourth group of macaques (group 4) who received subcutaneously the FTC and high-dose tenofovir used in group 3 only 2 h before and 24 h after each weekly virus challenge. A total of 18 control macaques did not receive any drug treatment. Of these macaques, nine were part of this study and nine were historical controls from earlier studies done under identical conditions in Rhesus macaques with the same virus stock, inoculum size, and inoculation protocol.

Oral administration of FTC and TDF was performed by mixing the drug powders with fruit (apples or bananas and infrequently oranges). Stability analysis for up to 24 h by high-pressure liquid chromatography (HPLC) demonstrated that TDF was stable in fresh apple, banana, or orange juice (96% in fruit compared to 98% in water; unpublished data). Macaques were observed to ensure they had taken the drugs. Overall daily drug ingestion was very high; animals missed only one to seven doses during the whole study period. Infected animal AG-81 (see below) missed five doses; one dose 14 d before and four sporadic doses after viral RNA was detected in plasma. Infected animal AI-54 only missed one dose 10 d before the first detectable RNA. Stock solutions of tenofovir and FTC were prepared in distilled water or phosphate-buffered saline, respectively, and injected subcutaneously [21,22].

Tenofovir disoproxil fumarate (9-[(R)-2[[bis[[isopropoxycarbonyl)oxy]-methoxy] phosphinyl]methoxy]propyl]adenine fumarate; TDF), tenofovir ((R)-9-(2-phosphonylmethoxypropyl) adenine; PMPA), and emtricitabine (5-fluoro-1-(2R,5S)-[2-(hydroxymethyl)-1,3-oxathiolan-5-yl]cytosine; FTC) were kindly provided by Gilead Sciences through a material transfer agreement.

### Macaque Model of Rectal SHIV Transmission

The efficacy of different PrEP modalities was evaluated using a repeated exposure macaque model of rectal transmission [12,19]. Rhesus macaques were exposed rectally once weekly for up to 14 wk to a SHIV_SF162P3_ chimeric virus that contains the tat, rev, and env coding regions of HIV-1_SF162_ in a background of SIVmac239 (National Institutes of Health [NIH] AIDS Research and Reference Reagent Program [23,24]). The SHIV162p3 challenge dose was 10 TCID_50_ or 7.6 × 10^5^ RNA copies, which is within the range of HIV-1 RNA levels in semen (10^3^–10^6^ copies/ml) during acute infection in humans and higher than the levels (10^2^–10^4^ copies/ml) seen after primary viremia [20,25]. Virus exposures were done 2 h after drug treatment by a non-traumatic inoculation of 1 ml of SHIV_SF162P3_ into the rectal vault via a sterile gastric feeding tube of adjusted length [19]. Macaques were anesthetized using standard doses of ketamine hydrochloride. Anesthetized macaques remained recumbent for at least 15 min after each intrarectal inoculation. Virus exposures were stopped when a macaque became SHIV RNA positive. All experiments were done under highly controlled conditions by the same personnel using the same virus stock, inoculum dose, and inoculation method.

The four study groups were staggered for logistic feasibility given the long follow-up (6.5 mo) in this model and to avoid unnecessary use of macaques. Group 3, which received the highest dose of FTC/tenofovir, was started before the FTC-only animals in group 1 because if this combination was not protective, testing FTC only would have not been pursued. Similarly, the intermittent FTC/tenofovir arm was done after data were available from the daily interventions. Four protected animals (three from group 3, one from group 1) were used again after a washout period of 8–12 mo; two were used in group 2 and two were used in group 4. Details on all the individual macaques, the regimens received, the sequence and outcome of each series, and the re-use of four animals are shown in Text S1 and Table S1. Blinding of the animal handlers was not done.

### Drug Doses

Small mammals usually eliminate drugs faster than larger mammals [26,27]. We therefore performed single-dose pharmacokinetic studies in Rhesus macaques to determine the FTC and TDF doses that achieve plasma and intracellular levels comparable to those seen clinically in humans. Six macaques were each given 48, 30, 20, 16, 15, and 12 mg/kg FTC by oral gavage; plasma and intracellular drug levels in peripheral blood mononuclear cells (PBMCs) were determined as described below at 1, 2, 4, 8, and 24 h. The area under the plasma concentration time curve (AUC) values over a 24 h interval increased linearly with the FTC dosing (unpublished data). The dose of 20 mg/kg FTC resulted in an AUC value comparable to that achieved in humans receiving 200 mg FTC (13.2 μg × h/ml and 10 ± 3.1 μg × h/ml, respectively) [17]. Intracellular FTC-triphosphate (FTC-TP) levels in this animal were essentially similar to those in humans [17]. FTC-TP levels were highest at 1 h (1,419 fmol/10^6^ cells), between 1,173–966 fmol/10^6^ cells from 2–8 h, and 273 fmol/10^6^ cells at 24 h. To assess oral bioavailability of FTC in macaques, two additional macaques received a dose of 11 or 17 mg/kg of FTC subcutaneously. Plasma FTC levels (AUC values of 6.1 and 12.2 μg × h/ml, respectively) were comparable to those achieved in the two macaques that received 12 or 16 mg/kg of FTC orally (7.9 and 11.2 μg × h/ml, respectively) indicating approximately 100% absorption. Therefore, we considered a once-daily oral or subcutaneous dose of 20 mg/kg of FTC as comparable to the dosing currently used in humans.

In humans, a wide range of tenofovir exposure is generally seen with once daily dosing of 300 mg of TDF (plasma AUC values of 2.3 ± 0.69 μg × h/ml; range, 2.1–3.2 μg × h/ml) [28]. We performed single-dose pharmacokinetic studies in five macaques that were each given by oral gavage a dose of 15, 20, 24, 37, and 46 mg/kg. Consistent with previous studies, we found a linear relationship between the dose of TDF and plasma AUC values. We also found that a dose of 20 or 24 mg/kg of TDF resulted in plasma tenofovir levels (AUC values of 3.2 and 3.4, respectively) that are within the range of those achieved in humans, while the other doses were outside this range (1.3, 4.1, and 5.8 μg × h/ml for 15, 37, and 46 mg/kg, respectively) [12,13,27,28]. Mean intracellular tenofovir-diphosphate (tenofovir-DP) levels measured between 1–4 h in these two macaques were 107 fmol/10^6^ cells (range, 60–169 fmol/10^6^ cells) and 248 fmol/10^6^ cells (range, 175–335 fmol/10^6^ cells), respectively, also comparable to the levels observed in humans 1–4 h after oral administration of 300 mg TDF (129–373 fmol/10^6^ cells) [16]. We therefore considered a once-daily dose of 22 mg/kg of TDF to be equivalent to the human dose.

### SHIV RNA Virus Load Assay and Amplification of Proviral Sequences

Plasma SHIV RNA was quantified using a real-time RT-PCR assay previously described [12]. This assay format has a sensitivity of 50 RNA copies/ml. RNA was extracted from virus pellets obtained by ultracentrifugation of 1 ml plasma. Prior to RNA extraction and to control for the efficiency of extraction, a known amount of virus particles (3 × 10^5^) from an HIV-1 CM240 virus stock was added to each plasma sample. Reverse transcription and PCR amplification of SHIV (gag) and HIV-1 (env) sequences was done using primers specific for SIVmac239 and HIV-1 CM240, respectively [12]. HIV-1 CM240 was obtained from the NIH AIDS Research and Reference Reagent Program. Amplification of proviral DNA from PBMCs was done as previously described using primers and probes specific for SIVmac239 pol [19].

### Detection of Genotypic Resistance by Standard and Sensitive Assays

FTC and tenofovir resistance was monitored by standard sequence analysis of SIV RT (551 bp; amino acids 52 to 234) and by sensitive allele-specific real-time PCR methods for the K65R and M184V mutations. Sequence analysis was done from plasma viruses using an RT-PCR procedure as previously described [12]. The Vector NTI program (version 7, 2001) was used to analyze the data and to determine deduced amino-acid sequences. Detection of low-frequency K65R and M184V mutants in plasma by real-time PCR was done as previously described [29]. A similar testing approach is used to assess minority drug-resistant HIV subpopulations in humans [30]. The sensitive SIV assays can detect 0.4% of K65R and 0.6% of M184V cloned sequences in a background of wild-type plasmid.

### Serology

Virus-specific serologic responses (IgG and IgM) were measured using a synthetic-peptide EIA (Genetic Systems HIV-1/HIV-2; BioRad) assay.

### Drug Levels in Plasma and in Peripheral Blood Mononuclear Cells

Tenofovir and FTC were extracted from 100 μl plasma by protein precipitation with 400 μl methanol containing 250 nM 3TC as an internal standard. Following precipitation, the solution was maintained at 4 °C for 10 min followed by vortexing for another 10 min. The supernatant was then evaporated to dryness in a heated vacuum centrifuge. Dry samples were stored at −20 °C until liquid chromatography–tandem mass spectrometry (LC-MS/MS) analysis. Drug levels were analyzed by using HPLC (LC Packings Ultimate 3000 modular LC System; Dionex) and ultra triple quadrupole mass spectrometer (Thermo Electron). Calibration curves were generated from standards of tenofovir and FTC by serial dilutions in human plasma over the range from 3.33 to 100,000 ng/ml. The lower limit of quantification was 3.33 ng/ml. All calibration curves had *r* ^2^ values greater than 0.99.

Tenofovir-DP and FTC-TP were extracted from macaque PBMCs using 60% methanol. Briefly, PBMCs were washed twice with phosphate-buffered saline and then resuspended in 1 ml of ice-cold methanol containing 4 nM 3TC-TP as an internal standard for both tenofovir-DP and FTC-TP. Samples were extracted overnight at −80 °C, centrifuged, and dried under a stream of air. Dried extracts were maintained at −80 °C until analysis. Prior to analysis, each sample was reconstituted in 100 μl 50 mM ammonium acetate buffer (pH 7.0) and centrifuged at 17,000*g* to remove insoluble particulates. Calibration curves were generated from standards of tenofovir-DP and FTC-TP by serial dilutions in blank human PBM extract (10^7^ cells/100 μl 50 mM ammonium acetate buffer) over the range from 0.25 to 10 nM for tenofovir-DP and 2.5 to 100 nM for FTC-TP. The lower limit of quantification was 0.25 nM for tenofovir-DP and 2.5 nM for FTC-TP. All calibration curves had *r* ^2^ values greater than 0.99. Drug levels were analyzed by HPLC (Agilent 1100 LC system; Agilent Technologies) with mass spectrometry detection (TSQ Quantum; Thermo Electron).

### Statistical Methods

The exact log-rank test was used to ascertain statistical difference between groups of macaques (treatment and control or two treatment groups). The Cox proportional hazards model was used to estimate the relative hazard ratio (HR), for a discrete-time survival analysis of the treatment and control groups, with use of the number of inoculations as the time variable. Though the sample sizes were small, model diagnostics supported the use of the Cox proportional hazards model. All statistical analyses for calculation of the efficacy of the different interventions were performed using SAS software (version 9.1; SAS Institute) and StatXact software (version 6.3; Cytel). For infected macaques, a linear mixed effects model was fit to the log_10_ of viral load with the macaques as a random effect and group (treatment or control) and time (weeks after peak viral load) as fixed effects.

## Results

### PrEP Efficacy of Daily FTC and FTC/TDF Combinations

**Figure 2:**
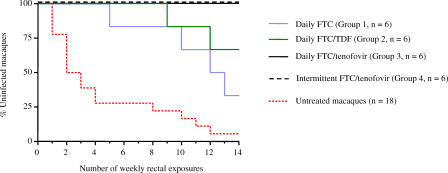
Protection against Repeated Rectal Virus Exposures by Daily or Intermittent PrEP Each survival curve represents the cumulative proportion of uninfected macaques as a function of the number of weekly rectal exposures. Protected animals in groups 1–4 remained negative after a mean washout out of 27 wk (range, 17–60 wk).

Figure 2 shows the rate of infection in animals that received PrEP and in 18 untreated control macaques based on the first detectable viral RNA in plasma. Untreated macaques were infected after a median of two rectal exposures (mean, four rectal exposures). This suggests that an animal receiving PrEP and remaining uninfected after 14 virus challenges would have been protected against a median of seven transmission events. The majority of control animals (13/18 or 72%) were infected during the first four challenges; four (22%) were infected between exposures 8 and 12, and only one macaque (6%) remained uninfected after 14 exposures. Proviral DNA was usually detected at the time of plasma RNA detection or within 1–2 wk thereafter. Virus-specific antibody responses were observed within 7 to 35 d (median, 21 d) after the first detectable RNA in plasma. Three control macaques had persistently high viral loads in plasma but remained seronegative. Animals in the PrEP arms were considered protected from systemic SHIV infection if they were seronegative and negative for SHIV plasma RNA and SHIV DNA in PBMCs during PrEP and the following 70 d of washout in the absence of any drug treatment [31].

All PrEP regimens offered some degree of protection. Of the six macaques in group 1 receiving FTC only, two were protected after 14 exposures, whereas four had detectable viral RNA at exposures 5 (AG-80), 10 (AG-46), 12 (AH-04), and 13 (AG-07); infection in these four macaques was delayed compared to untreated controls (*p* = 0.004, exact log-rank test). These data demonstrate the prophylactic activity of FTC. Infection was also confirmed by DNA-PCR for provirus in PBMC and seroconversion observed 3, 1, 2, and 6 wk after the first detectable RNA, respectively. To compare the temporal probability of risk for SHIV infection in controls relative to treated animals, Cox proportional HRs were calculated. Compared to controls, animals receiving FTC had a 3.8-fold lower risk of infection (Cox HR, 3.8, *p* = 0.02).

More protection was seen in group 2 (oral FTC and TDF); four of the six macaques were protected and only two (AG-81 and AI-54) were infected, at exposures 9 and 12. Both infections were significantly delayed from controls (*p* = 0.0004, exact log-rank test). These two macaques were seropositive 2 wk after the first detectable viral RNA in plasma and both were proviral DNA positive at weeks 10 and 12, respectively. Risk of infection in group 2 macaques receiving FTC and TDF was significantly reduced by 7.8-fold compared to controls (Cox HR, 7.8, *p* = 0.008).

### Breakthrough Infections Have Blunted Wild-Type Viremias

**Figure 3:**
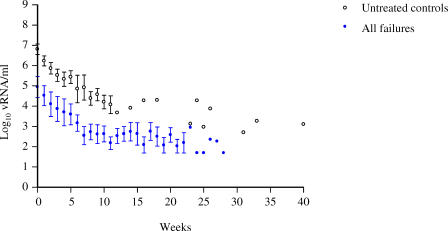
Breakthrough Infections during PrEP Show Blunted Acute Viremias Each time point represents the mean virus load observed in treated (*n* = 6) or untreated control (*n* = 10) macaques. Time 0 indicates the peak plasma virus load. Bars denote the standard error of the mean.

**Figure 4:**
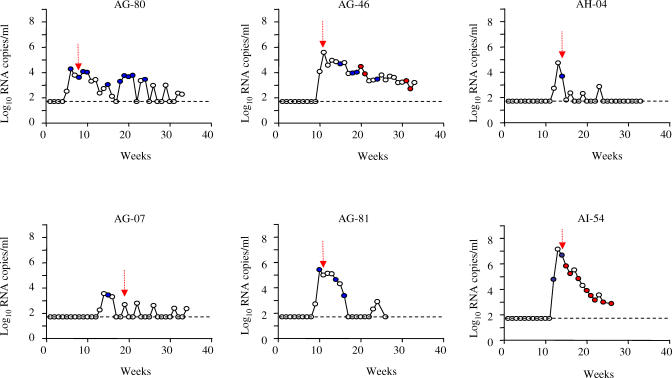
Dynamics of Infection in Animals Infected during PrEP with FTC (AG-80, AG-46, AH-04, and AG-07) or FTC/TDF (AG-81 and AI-54) Red circles indicate detectable M184V/I mutation; wild-type sequences are shown in blue. Open circles indicate time points that were not genotyped. The first detectable antibody response is shown as red arrows. The broken line denotes the limit of detection of the virus load assay (50 copies/ml).

When PrEP fails to completely prevent transmission, the antiretroviral regimen may select for viruses with resistance to the drugs [32]. We therefore continued drug treatment in the six animals that failed PrEP in groups 1 and 2 and followed virus load dynamics and drug resistance emergence. Differences were first noted in acute viremia between breakthrough and control infections. Figure 3 compares virus loads in the breakthrough infections and 10 untreated macaques with sufficient longitudinal data. The mean peak viremia in the six treated macaques was 4.9 ± 0.5 log_10_ RNA copies/ml, 2.0 log_10_ lower than in untreated controls (6.9 ± 0.3 log_10_ RNA; *p* = 0.007, Mann-Whitney test; Figure 3). This difference was maintained up to week 11 as virus loads declined at similar rates in both groups (−0.24 ± 0.02 log_10_/wk and −0.24 ± 0.03 log_10_/wk respectively; *p* = 0.99). Figure 4 shows that three FTC and one FTC/TDF failures had the first undetectable virus loads as early as weeks 3 (AG-07), 4 (AH-04), 7 (AG-81), and 11 (AG-80) after the peak viremia. All four animals maintained low or undetectable virus loads for up to 20 wk. In contrast, all 10 untreated macaques had detectable virus loads during a median follow-up period of 7 wk (range, 5–40 wk; Figure 3).

Sequence analysis of SIV RT showed that all six breakthrough infections were initiated with wild-type virus. Four animals had no evidence of drug resistance by both sensitive and conventional testing despite extended (median, 23 wk; range, 13–29 wk) treatment. Two animals had M184V (AG-46; FTC-treated) or M184I (AI-54; FTC/TDF-treated) mutations associated with FTC resistance 10 and 3 wk after the first detectable RNA, respectively (Figure 4). The tenofovir-associated K65R mutation was not detected in the two animals receiving FTC/TDF.

### Full Protection by Daily or Intermittent PrEP

In an effort to completely block SHIV transmission, we administered subcutaneously FTC (20 mg/kg) and a higher dose of tenofovir (22 mg/kg) to a third group of six macaques (group 3). All of the six macaques that received this daily PrEP regimen remained uninfected after 14 exposures (*p* = 0.00005 compared to untreated controls, exact log-rank test; Figure 2). We also assessed if PrEP given intermittently around each virus exposure is equally protective to daily PrEP. A group of six macaques was injected subcutaneously with this highly effective regimen only 2 h before and 24 h after each weekly rectal challenge. All six animals received 14 weekly challenges, and all were found to be protected from infection (Figure 2). None of the animals were seropositive or had detectable viral sequences either in plasma or PBMCs at any of the weekly time points during the 14-wk intermittent PrEP period and up to 70 d thereafter.

## Discussion

In this study, we investigated the relationship between PrEP antiviral activity and protection by using a repeat-exposure macaque model that closely resembles human transmission. At dosing equivalent to that used in humans, we found that daily FTC was partially protective, and that the addition of TDF increased effectiveness. We also show that subcutaneous FTC and high-dose tenofovir completely blocked rectal transmission. These findings show that full protection against repeated exposures by daily PrEP is possible in a primate model, and that PrEP effectiveness correlates with increases in antiviral activity.

None of the protected animals showed any evidence of transient systemic infection, likely reflecting the ability of FTC and tenofovir to effectively block the earliest SHIV infections, possibly at the mucosal point of entry, before significant systemic dissemination of the virus occurs. Since inhibiting virus establishment shortly after exposure may be critical to PrEP efficacy, we also explored if PrEP given intermittently around each virus exposure could be equally protective to daily PrEP. Earlier studies in newborn macaques exposed orally to a highly virulent SIVmac251 strain have shown the promise of short PrEP interventions [33,34]. Intermittent PrEP modalities are highly desirable because of their convenience, potential cost-effectiveness, and lower risks of drug toxicity. Both FTC and tenofovir have long (40 to >60 h) intracellular half-lives in humans [16–18] suggesting the possibility of extended prophylactic activity when administered around virus exposures. We found FTC/tenofovir given intermittently as a two-dose PrEP around each of 14 weekly virus exposures to be as fully protective as the same regimen given daily. Therefore, intermittent PrEP with potent regimens are highly promising modalities. Evaluation of intermittent PrEP with different drug combinations, possibly of different classes, and defining minimal dose requirement and optimal timing relative to virus exposure will all be important.

While many biologic similarities exist between rectal and vaginal HIV transmission, some differences in the early events of mucosal infection and dissemination kinetics are possible [8,35]. Therefore, it is important to confirm the PrEP efficacy of these regimens against vaginal transmission in appropriate macaque models. Although daily PrEP with a Truvada-equivalent dosing was highly effective, a regimen with more tenofovir was required to completely block transmission in this model. However, the dose of tenofovir in this regimen would likely be toxic in humans. More work in macaque models could possibly identify two- or three-drug combinations that are fully protective and yet carry low risks of toxicity. The increasing availability of new drugs in different classes such as those that block viral integration or entry through CCR5 will provide additional possibilities. The results of such animal studies may help guide designs of clinical trials that will ultimately measure effectiveness of various PrEP regimens against sexual HIV transmission.

Initial macaque studies with tenofovir used single and non-physiologic doses of SHIV or SIV (10^3^ to 10^5^ TCID_50_) capable of yielding high infection rates in untreated controls [10,11,33,34]. We used a more physiological virus dose that fell within the upper range of viral load observed in human semen during acute HIV-1 infection [20]. The repeated nature of the model has also the advantage of evaluating protection over multiple transmission events. The infection of control macaques after a median of two exposures suggests that treated animals that remain uninfected after 14 challenges were protected over a median of seven transmission events. This model maintains high stringency, increases statistical power, provides improved estimations of risk reductions, and reduces the number of macaques [36].

Although a repeat-challenge model is more relevant to human transmission, which typically requires multiple exposures, a disadvantage of the model is that it is logistically demanding over a long period of time. This limits the ability to do multiple concurrent arms, which raises a potential for bias. However, animal studies with non-concurrent arms can be well controlled. In our study, we have staggered interventions because of logistic feasibility and to prevent unnecessary use of animals. All animal procedures were done under identical conditions by the same personnel and experimental protocol, thus minimizing the potential for bias. Likewise, it is not known if repeated exposures to the virus can ultimately alter susceptibility to infection. The similar infection rates observed among previously or newly exposed animals suggest that the impact of repeated exposures on susceptibility to infection is minimal [12,37].

Several important observations were made from the longitudinal analysis of the breakthrough infections. The finding that wild-type SHIV initiated all six infections suggests that PrEP failure in these animals is due to residual virus replication in cells not protected by drugs, rather than a rapid selection of a drug-resistant virus. Of the four animals infected during FTC treatment, only one selected resistant viruses, while an FTC-resistant virus emerged in one of two animals that failed FTC/TDF PrEP. The absence of tenofovir resistance in both macaques is consistent with clinical observations showing resistance to FTC and not tenofovir as the most frequent pathway of resistance to Truvada [38]. The two macaques in which FTC-resistant mutants emerged had the highest peak viremias, suggesting that selection of drug resistance may be facilitated by higher virus replication. Thus, lower acute viremias may have diminished the risk of resistance during extended treatment with FTC or FTC/TDF. Similar blunted viremias during early infection have been noted in macaques failing PrEP with an orally administered CCR5 inhibitor [39]. These data underscore the potential differences in virus load and drug resistance dynamics during PrEP failures from those in mono- or dual drug therapy of established infections [21,22,40,41].

The attenuated acute viremias may have additional clinical and public health implications. It is well established that massive depletion of CD4^+^ T cells, specifically CD4^+^ memory T cells, begins in the acute stage of SIV as well as HIV-1 infection in the gut and other lymphoid tissues, and is generally proportional to the degree of virus replication [42–44]. Substantial reductions in acute viremia may conceivably reduce CD4^+^ T cell depletion, help preserve immune function, and attenuate the course of HIV infection. In humans, reduction in virus set points by 1 log_10_ have been estimated to double the time to progression to HIV disease [45]. Reductions in virus loads in the animals that failed PrEP were apparent at the first time points before seroconversion and were sustained under continued drug treatment after all the animals seroconverted. Blunted viremia during acute infection in persons who fail PrEP will likely depend on the period of drug exposure and the potency of the PrEP regimen. PrEP-treated populations will likely be monitored by serologic testing for infection to minimize drug exposure and reduce the risks of drug resistance. The period of drug exposure will thus depend on the frequency of serologic testing but will inevitably be at least several weeks long, enough to affect early CD4^+^ T cell depletion. Individuals with acute infections who have very high virus loads may also play a key role in the epidemic spread of HIV-1 because they are more infectious than individuals with chronic infections who have lower virus loads [46–48]. Therefore, reductions in acute viremia during PrEP treatment may contribute to decreases in HIV-1 transmissibility at the population level and could add to the overall effectiveness of PrEP.

The data from this study demonstrate the potential high effectiveness of daily or intermittent PrEP against sexual HIV transmission, support expanding PrEP trials in humans and identify promising PrEP modalities.

## Supporting Information

Alternative Language Abstract S1 Translation of the Abstract into French by Dominique Rollin and Walid Heneine (23 KB DOC)

Alternative Language Abstract S2 Translation of the Abstract into Spanish by J. Gerardo García-Lerma (43 KB DOC)

Table S1 Macaques, Interventions, and Outcome of the Challenge Series (51 KB DOC)

Text S1 Description of Current and Historic Macaques and Susceptibility to Infection (28 KB DOC)

## Abbreviations

AUC: area under the curve
FTC: emtricitabine
FTC-TP: FTC-triphosphate
HR: hazard ratio
PBMC: peripheral blood mononuclear cell
PrEP: pre-exposure prophylaxis
RT: reverse transcriptase
SIV: simian immunodeficiency virus
TDF: tenofovir–disoproxil fumarate
TFV-DP: tenofovir-diphosphate
