# Supplementary material for: Prevention of Rectal SHIV Transmission in Macaques by Daily or Intermittent Prophylaxis with Emtricitabine and Tenofovir
Source: PLoS Med. 2008 Feb 5;5(2):e28. doi: 10.1371/journal.pmed.0050028 (PMC2225435; doi:10.1371/journal.pmed.0050028)
Supplement: Supplementary file 1 [file pmed.0050028.sd001.doc]

Prevention de la transmission rectale du virus de l’immunodéficience simienne chez les Macaques par une prophylaxie quotidienne ou intermittente avec l’emtricitabine et le tenofovir

**Résumé**

**Objectifs:** En l’absence d’un vaccin efficace, le VIH continue à se propager globalement, soulignant l’importance du besoin de stratégies nouvelles pour en limiter la transmission. La prophylaxie pré-exposition (PprE) avec des médicaments antirétroviraux pourrait être une intervention stratégique efficace si des modalités de PprE hautement protectrices et d’un bon rapport côut-efficacité pouvaient être définies. Nous avons évalué des PprE d’activité antivirale croissante administrées quotidiennement ou de façon intermittente à un modèle macaque qui se rapproche de la transmission chez l’homme.

**Méthodes et résultats**: Nous avons utilisé un modèle macaque avec exposition répétée consistant en 14 infections hebdomadaires rectales. Trois traitements médicamenteux ont été administrés une fois par jour à 3 groupes de 6 macaques rhésus. Le groupe 1 a reçu par voie sous-cutanée une dose d’emitricitabine (FTC) équivalente à la dose chez l’homme, le groupe 2 a reçu par voie orale une dose mixte de FTC et de fumarate de ténofovir disoproxil équivalente à la dose chez l’homme, et le groupe 3 a reçu par voie sous-cutanée la même dose de FTC et une dose plus élevée de tenofovir. Un quatrième groupe (groupe 4) a reçu de façon intermittente une PprE similaire à celle du groupe 3 seulement 2 heures avant et 24 heures après l’infection expérimentale hebdomadaire par le virus. Les résultats ont été comparés à 18 macaques contrôles qui n’ont reçu aucun traitement médicamenteux. Le risque d’infection chez les macaques traités des groupes 1 et 2 a été 3,8 et 7,8 fois plus bas que chez les macaques non traits (p=0,02 et p=0,008 respectivement). Les six macaques du groupe 3 ont tous été protégés. Les infections qui sont apparues malgré le traitement antirétroviral ont emoussé la virémie aigue et une résistance aux médicaments a été notée chez 2 des 6 animaux infectés. Tous les animaux du groupe 4 ayant reçu une PprE intermittente ont été protégés.

**Conclusions:** Ce modèle suggère qu’une PprE quotidienne consistant en un seul médicament antirétroviral peut être protectrice mais que la combinaison de 2 médicaments élève le niveau de protection. Une PprE intermittente, brève mais puissante, peut être aussi protectrice qu’une PprE quotidiennement. Ces résultats encouragent la réalisation d’essais cliniques chez l’homme et définissent des modes de PprE des plus prometteurs.
