# Supplementary material for: Prevention of Rectal SHIV Transmission in Macaques by Daily or Intermittent Prophylaxis with Emtricitabine and Tenofovir
Source: PLoS Med. 2008 Feb 5;5(2):e28. doi: 10.1371/journal.pmed.0050028 (PMC2225435; doi:10.1371/journal.pmed.0050028)
Supplement: Supplementary file 2 [file pmed.0050028.sd002.doc]

**Resumen**

**Introducción:** La continua expansión global del VIH y la ausencia de una vacuna efectiva acentúan la necesidad de identificar nuevas intervenciones biomédicas que reduzcan la transmisión del VIH. La profilaxis pre-exposición (PrEP) con drogas antirretrovirales puede suponer una estrategia de prevención efectiva siempre que se identifiquen modalidades eficaces y coste-efectivas. En este estudio se han investigado regímenes de PrEP diarios e intermitentes con distinta actividad antiviral empleando un modelo animal de transmisión del VIH en humanos.

**Métodos y resultados:** Se empleó un modelo animal de exposición semanal al virus por vía rectal hasta un total de 14 semanas. Tres regímenes de PrEP se administraron una vez al día a grupos de seis macacos. El primer grupo (grupo 1) recibió por vía subcutánea una dosis de emtricitabina (FTC) equivalente a la dosis empleada en humanos. El segundo grupo (grupo 2) recibió por vía oral FTC y fumarato de disoproxilo de tenofovir a dosis equivalentes a la dosis humana. El tercer grupo (grupo 3) recibió por vía subcutánea la misma dosis de FTC y una dosis mayor de tenofovir. Un cuarto grupo (grupo 4) recibió de forma intermitente la dosis de FTC y tenofovir empleada en el grupo 3 solamente dos horas antes y 24 horas después de cada exposición semanal al virus. Los resultados se compararon con 18 macacos que no recibieron ningún tratamiento. El riesgo de infección en macacos de los grupos 1 y 2 fue 3,8 y 7,8 veces menor que en macacos no tratados (p = 0,02 y p = 0,008, respectivamente). Ninguno de los seis macacos del grupo 3 se infectó. Los animales infectados en los grupos 1 y 2 tenían viremias bajas; aparición de resistencia a drogas se observó en dos de los seis animales. Todos los macacos del grupo 4 que recibieron PrEP de una manera intermitente fueron protegidos de la infección.

**Conclusiones:** El modelo animal sugiere que la PrEP diaria con una única droga puede tener un efecto protector pero que la combinación de drogas puede ser necesaria para aumentar el nivel de protección. Intervenciones de PrEP cortas y potentes pueden también proteger de la infección. Estos resultados apoyan la realización de ensayos clínicos de PrEP en humanos e identifican las modalidades más prometedoras.
