# Supplementary material for: Prevention of Rectal SHIV Transmission in Macaques by Daily or Intermittent Prophylaxis with Emtricitabine and Tenofovir
Source: PLoS Med. 2008 Feb 5;5(2):e28. doi: 10.1371/journal.pmed.0050028 (PMC2225435; doi:10.1371/journal.pmed.0050028)
Supplement: Supplementary file 3 [file pmed.0050028.sd003.doc]

**Text S1 - Supplementary information**

The data reported in this manuscript were generated from a total of 33 macaques. Six macaques were in each of the 4 treatment groups and 9 were controls. Four previously-treated and protected animals were used again ~8-12 months after the last virus challenge; 2 were used in the oral truvada group (group 2) and 2 in the intermittent FTC/tenofovir group (group 4) (Table S1). Previous data have shown no difference in susceptibility to infection or virus loads between newly exposed or previously exposed macaques that were protected from repeat challenges by tenofovir or a microbicide containing cellulose acetate phthalate. Subsequent challenges in these previously-exposed macaques showed infections that were indistinguishable from unexposed animals [1,2]. After the completion of the study, one of 4 re-used animals (AG-65 from group 3) was challenged along with 3 other exposed and protected animals (79R, 17V, and 68-0) from group 4. These 4 macaques were infected at challenges 1, 1, 2 and 3, as commonly seen with newly-exposed animals. These data establish the susceptibility of these animals to infection and show that it is not different from newly-exposed animals. To further demonstrate that these 4 animals have no impact on our conclusion, we re-analyzed the data excluding the two macaques from group 2 (uninfected AH-03 and AG-81 infected at challenge 9) and the two macaques from the intermittent FTC/tenofovir group (uninfected AG-45 and AG-65). The protection achieved by daily truvada in group 2 and intermittent PrEP both remained statistically different from controls; log-rank p = 0.0012 (Cox proportional hazard (PH) = 11.1, p= 0.022) for group 2, and log-rank p = 0.00068 for intermittent PrEP. The Cox PH value for group 2 macaques (11.1) was not statistically different from the previous value of 7.8.

Data from 18 controls are used in the study; 9 were part of this study and 9 were historical controls from earlier studies done under identical conditions in Rhesus macaques with the same virus stock, inoculum size, and inoculation protocol. These 9 controls animals were infected in 2004 (6 macaques) and early (2 macaques) or late 2005 (1 macaque). We reanalyzed the protection achieved by all interventions using only 9 current controls. Overall the conclusion remained unchanged. The protection by Truvada (group 2), FTC/TFV (group 3), and intermittent FTC/TFV (group 4) remained significant (log-rank p value p<0.05) and close to significant with the weaker FTC-only intervention (group 1, log-rank p value p = 0.07).

**References**

1. Kim CN, Adams DR, Bashirian S, Butera S, Folks TM, et al. (2006) Repetitive exposures with simian/human immunodeficiency viruses: strategy to study HIV pre-clinical interventions in non-human primates. J Med Primatol 35: 210-216.

2. Subbarao S, Otten RA, Ramos A, Kim C, Jackson E, et al. (2006) Chemoprophylaxis with tenofovir disoproxil fumarate provided partial protection against infection with simian human immunodeficiency virus in macaques given multiple virus challenges. J Infect Dis 194: 904-911.
