# Supplementary material for: Prevention of Rectal SHIV Transmission in Macaques by Daily or Intermittent Prophylaxis with Emtricitabine and Tenofovir
Source: PLoS Med. 2008 Feb 5;5(2):e28. doi: 10.1371/journal.pmed.0050028 (PMC2225435; doi:10.1371/journal.pmed.0050028)
Supplement: Supplementary file 4 [file pmed.0050028.st001.doc]

**Table S1. Macaques, interventions, and outcome of the challenge series**

| Macaques | Pre challenge drug treatment | Initiation of challenges | Infection outcome | Last challenge | Study  Completion |
| --- | --- | --- | --- | --- | --- |
| Group 3 (subcut FTC/TFV)  510  AH-03  AG-65  AG-74  AG-81  AG-82  Untreated  AG-26  AG-57  AG-66  AG-47  AG-44  AG-63  Group 1 (subcut FTC)  AG-07  AG-46  AG-80  AG-45  AG-76  AH-04  Group 2 (Oral FTC/TDF)  **AH-03**  AJ-05  AI-22  AI-54  AH-67  **AG-81**  Untreated  07-Z  AI-98  21-Z  Group 4 (intermittent FTC/TFV)  79-R  17-V  64-V  68-0  **AG-45**  **AG-65** | 5/23/05  5/23/05  5/23/05  5/23/05  5/23/05  5/23/05  -  -  -  -  -  -  11/4/05  11/4/05  11/4/05  11/4/05  11/4/05  11/4/05  3/24/06  3/24/06  3/24/06  3/24/06  3/24/06  3/24/06  -  -  -  n.a.  n.a.  n.a.  n.a.  n.a.  n.a. | 6/1/05  6/1/05  6/1/05  6/1/05  6/1/05  6/1/05  6/1/05  6/1/05  6/1/05  6/1/05  6/1/05  6/1/05  11/14/05  11/14/05  11/14/05  11/14/05  11/14/05  11/14/05  4/5/06  4/5/06  4/5/06  4/5/06  4/5/06  4/4/06  4/5/06  4/5/06  4/5/06  9/12/06  9/12/06  9/12/06  9/12/06  9/12/06  9/12/06 | Uninfected  Uninfected  Uninfected  Uninfected  Uninfected  Uninfected  Infected (exposure 4)  Infected (exposure 3)  Infected (exposure 2)  Infected (exposure 2)  Infected (exposure 10)  Uninfected  Infected (exposure 13)  Infected (exposure 10)  Infected (exposure 5)  Uninfected  Uninfected  Infected (exposure 12)  Uninfected  Uninfected  Uninfected  Infected (exposure 12)  Uninfected  Infected (exposure 9)  Infected (exposure 4)  Infected (exposure 3)  Infected (exposure 12)  Uninfected  Uninfected  Uninfected  Uninfected  Uninfected  Uninfected | 8/31/05  8/31/05  8/31/05  8/31/05  8/31/05  8/31/05  6/22/05  6/15/05  6/8/05  6/8/05  8/3/05  8/31/05  2/6/06  1/16/06  12/12/05  2/13/06  2/13/06  1/30/06  7/5/06  7/5/06  7/5/06  6/21/06  7/5/06  5/31/06  4/26/06  4/19/06  6/21/06  12/12/06  12/12/06  12/12/06  12/12/06  12/12/06  12/12/06 | 12/7/05  12/7/05  12/7/05  12/7/05  12/7/05  12/7/05  12/7/05  12/7/05  12/7/05  12/7/05  12/7/05  12/7/05  5/22/06  5/22/06  5/22/06  5/22/06  5/22/06  5/22/06  10/11/06  10/11/06  10/11/06  10/11/06  10/11/06  10/11/06  10/11/06  10/11/06  10/11/06    2/20/07  2/20/07  2/20/07  2/20/07  2/20/07  2/20/07 |

n.a.; not applicable, bold; reused animals

Historic controls: infection at exposure 1, 1, 1, 1, 2, 2, 2, 8, and 11

**References**

1. Kim CN, Adams DR, Bashirian S, Butera S, Folks TM, et al. (2006) Repetitive exposures with simian/human immunodeficiency viruses: strategy to study HIV pre-clinical interventions in non-human primates. J Med Primatol 35: 210-216.

2. Subbarao S, Otten RA, Ramos A, Kim C, Jackson E, et al. (2006) Chemoprophylaxis with tenofovir disoproxil fumarate provided partial protection against infection with simian human immunodeficiency virus in macaques given multiple virus challenges. J Infect Dis 194: 904-911.
